# Outlier Detection in GNSS Pseudo-Range/Doppler Measurements for Robust Localization

**DOI:** 10.3390/s16040580

**Published:** 2016-04-22

**Authors:** Salim Zair, Sylvie Le Hégarat-Mascle, Emmanuel Seignez

**Affiliations:** SATIE (Systems Applications of Information Energy Technologies) laboratory, University of Paris-Sud, 91405 Orsay, France; sylvie.le-hegarat@u-psud.fr (S.L.H.-M.); emmanuel.seignez@u-psud.fr (E.S.)

**Keywords:** Global Navigation Satellite Systems (GNSS), robust localization, *a contrario* decision, particle filter, Rao-Blackwellization

## Abstract

In urban areas or space-constrained environments with obstacles, vehicle localization using Global Navigation Satellite System (GNSS) data is hindered by Non-Line Of Sight (NLOS) and multipath receptions. These phenomena induce faulty data that disrupt the precise localization of the GNSS receiver. In this study, we detect the outliers among the observations, Pseudo-Range (PR) and/or Doppler measurements, and we evaluate how discarding them improves the localization. We specify *a contrario* modeling for GNSS raw data to derive an algorithm that partitions the dataset between inliers and outliers. Then, only the inlier data are considered in the localization process performed either through a classical Particle Filter (PF) or a Rao-Blackwellization (RB) approach. Both localization algorithms exclusively use GNSS data, but they differ by the way Doppler measurements are processed. An experiment has been performed with a GPS receiver aboard a vehicle. Results show that the proposed algorithms are able to detect the ‘outliers’ in the raw data while being robust to non-Gaussian noise and to intermittent satellite blockage. We compare the performance results achieved either estimating only PR outliers or estimating both PR and Doppler outliers. The best localization is achieved using the RB approach coupled with PR-Doppler outlier estimation.

## 1. Introduction

The Global Navigation Satellite Systems (GNSS), such as the Global Positioning Systems (GPS), have been developed to provide an absolute location on an Earth-Centered Earth-Fixed (ECEF) [1]. These sensors became very popular for autonomous navigation [2] and applications of Intelligent Transportation Systems (ITS) thanks to the worldwide coverage of these constellations and the rather low cost of the receivers. Even if several works have proposed to combine GNSS data with other information sources, either sensors (e.g., Inertial Measurement Unit (IMU) [3]) or prior information (e.g., maps [4,5]), there is still a need to improve the performance of GNSS-only localization. Indeed, even in the perspective of fusion with other data, the accuracy of the GNSS estimation will impact the location result. Then, this study focuses on GNSS-only localization.

Early works estimated the receiver location based on GNSS Pseudo-Range (PR) data. Recently, the estimation of the instantaneous velocity that may be derived from Doppler measurements has been proposed. For instance, [6] introduces both the PR and Doppler measurements in the Extended Kalman Filter (EKF). These Doppler measurements may be particularly helpful in constrained environments where the number of usable observations may drop. Indeed, in space-constrained areas, the obstacles (buildings, trees) reflect the signals sent by the satellites, inducing Non-Line Of Sight (NLOS) and multipath receptions. The corrupted measurements are characterized by a positive bias that increases the estimated satellite-receiver distance in a faulty way, which is difficult to model and to correct. The works in [7,8] have experimentally shown that Doppler measurements are affected, as well, although to a lesser extent than PR measurements, by multipaths and NLOS.

To detect the faulty data, a first approach is to analyze the signal measurements. For instance, [9] exploits the carrier to noise density measurement ($C/N_{0}$) in order to partition the observation set between LOS signals ($C/N_{0}\;≃45$ dB) and NLOS signals ($C/N_{0}\;\leq 40$ dB). However, in urban canyons, the NLOS signals may be stronger than the LOS one [8]. A second approach is to look toward robust estimation, *i.e*., methods that are able to cope with some faulty data. In GPS-based localization, we can cite the Receiver Autonomous Integrity Monitoring (RAIM) [10] or *q*-relaxation technique used in interval analysis [11]. Both approaches assume a bounded number of outliers. Assuming Gaussian noise, [12] proposed an EKF with outlier detection. The Particle Filter (PF), which has been proposed to resolve non-linear/non-Gaussian problems [13], was applied in [14] having discarded the outliers from the set of observations, whereas in [15], it was used to estimate both the corrupted bias on PR observations and the localization parameters. However, neither [15] nor [14] investigated the presence of outliers in Doppler measurements. Besides, [15] only considers simulated data.

The main contribution of this work is to propose a robust localization process that uses both PR and Doppler measurements. It is based on the adaptation of signal processing methods previously applied to other problems or data. It involves two parts: (i) the inlier/outlier partitioning characterized by the absence of a threshold; (ii) the filtering for a GPS-based localization characterized by its robustness to noise and to intermittent satellite blockage. The first point is achieved by formulating the problem in terms of minimization of a criterion, namely the Number of False Alarms (NFA). This criterion was introduced by [16,17] to measure the degree of surprise or contradiction of a structured observation relative to a noise (unstructured data) model, and it has been successfully applied to various problems in image processing [18,19,20,21,22]. In a previous work [23], we have defined and compared two NFA criteria, and we have shown that they are more efficient than classic statistical tests to partition the PR measurements between a consistent dataset (the inliers) against an inconsistent dataset (the outliers). However, in this first work, only PR measurements were considered. Then, this study develops the ideas and first results presented in conference paper [24], where a rather simple implementation of PF was considered. Based on temporal redundancy, the PF allows us to filter the noise present in the inlier data. However, for practical reasons, it cannot handle the state vector of large dimensionality. In this work, we propose to use a more sophisticated filter, namely the Rao–Blackwell Particle Filter (RBPF) [25]. Its principle is to split the state system into two subsystems, a linear part and a non-linear one, so that the linear part may be analytically solved, whereas the non-linear part is approximated using the importance sampling technique (like in PF). RBPF has already been applied successfully for navigation [26], tracking [27] and GPS multipath estimation [15]. In [26], RBPF was applied for GPS-based localization in urban canyons. However, the authors only consider PR measurements, whereas in this study, we propose to extend their filter to both PR and Doppler measurements and to couple it with the outlier detection using NFA criterion based on *a contrario* modeling. Concerning the application, we focus on land vehicle navigation in constrained environments. Then, to achieve similar localization performance in such environments as in open areas, our algorithm should be robust until about 40% of outliers. The “raw” data we consider (and among which outliers will be searched) are the PR and the Doppler measurements provided by the GNSS receiver ($L_{1}$ carrier). Even if they are already estimations from the pseudo-random codes, we call them “raw” by contrast to positioning values also provided by the GNSS receiver.

Section 2 introduces the notations and basic equations inherent to the localization problem using GNSS data. Section 3 describes the proposed method that involves a detection of outlier measurements in the dataset using the NFA criterion followed by the localization process using GPS raw data, either based on PF or on the RBPF algorithm. The experiment and related results are discussed in Section 4. Section 5 reports our conclusions and perspectives.

## 2. Problem Formulation

### 2.1. Observation Model

In this study, we consider two pieces of information provided by GNSS satellite $S_{j}$. The first one is the pseudo-range $\rho_{j}$ that is related to the distance between the receiver and $S_{j}$. Denoting by upper-script ${}^{⊺}$ the transpose operator, the receiver position is denoted $x_{r}={\left(e_{r},n_{r},u_{r}\right)}^{⊺}$ in the ENU (East, North, Up) coordinate local system, and the $S_{j}$ position is denoted $x_{S_{j}}={\left(e_{S_{j}},n_{S_{j}},u_{S_{j}}\right)}^{⊺}$. We choose the ENU frame for its wide use in land navigation (since it allows us to process the ‘up’ coordinates separately). Then, the pseudo-range depends on $x_{r}$, $x_{S_{j}}$, $\delta_{t}$ the time bias (difference) between the two unsynchronized clocks of the satellite and receiver, respectively, *c* the light velocity and random noise $\epsilon_{j}$:

(1) $$\begin{array}{ccc} \rho_{j} & = & ∥x_{r}-x_{S_{j}}∥+c\delta_{t}+\epsilon_{j},\\ & = & \sqrt{{\left(e_{r}-e_{S_{j}}\right)}^{2}+{\left(n_{r}-n_{S_{j}}\right)}^{2}+{\left(u_{r}-u_{S_{j}}\right)}^{2}}+c\delta_{t}+\epsilon_{j} \end{array}$$

Equation (1) is the simplest version of the PR observation equation. It does not represent multipath or NLOS receptions, so that they can be detected as deviations relatively to this model.

The second information piece is the Doppler measurement that is related to the receiver velocity ${\dot{x}}_{r}={\left({\dot{e}}_{r},{\dot{n}}_{r},{\dot{u}}_{r}\right)}^{⊺}$. Denoting ${\dot{x}}_{S_{j}}={\left({\dot{e}}_{S_{j}},{\dot{n}}_{S_{j}},{\dot{u}}_{S_{j}}\right)}^{⊺}$, the $S_{i}$ satellite velocity that is determined using broadcast ephemeris [28],

(2) $$\dot{\rho_{j}}=\left({\dot{x}}_{r}-{\dot{x}}_{S_{j}}\right)\cdot a_{j}-c{\dot{\delta }}_{t}+\epsilon_{j}^{\prime }$$

where $\dot{\rho_{j}}$, called the “PR rate”, is equal to $c\frac{D_{j}}{f_{1}}$, with $f_{1}=1.575$ GHz and $D_{j}$ the Doppler observation (in Hz) provided by $S_{j}$, $a_{j}$ is to the unit vector collinear to the straight line through the receiver and satellite $S_{j}$ ($a_{j}=\frac{x_{S_{j}}-x_{r}}{∥x_{S_{j}}-x_{r}∥}$), “·” denotes the dot product, ${\dot{\delta }}_{t}$ the clock drift and $\epsilon_{j}^{\prime }$ random noise.

### 2.2. Localization Problem

For location estimation, different systems of equations may be considered depending on the used data: PR, Doppler measurements or both data.

Firstly, only using PR, at least four observations are required to estimate vector $x_{r}$ and time bias $\delta_{t}$ by solving the system of Equation (1).

Secondly, only using Doppler measurements, *theoretically* vector ${\dot{x}}_{r}$, time drift ${\dot{\delta }}_{t}$ and vector $x_{r}$ could be estimated, since they all appear in Equation (2). However, two hindrances to this approach are: (i) per epoch, at least seven observations from different satellites would be required, which is incompatible with robustness to satellite blockage in constrained environments; (ii) Equation (2)’s sensitivity to $x_{r}$ is rather poor, since $x_{r}$ is involved only through $a_{j}$. Thus, practically, PR measurements are also used to derive an estimation of $x_{r}$ and then an estimation of $a_{j}$, denoted ${\tilde{a}}_{j}$, which is introduced in Equation (2):

(3) $$\dot{\rho_{j}}+{\dot{x}}_{S_{j}}\cdot {\tilde{a}}_{j}={\dot{x}}_{r}\cdot {\tilde{a}}_{j}-c{\dot{\delta }}_{t}+\epsilon_{j}^{\prime }$$

The third and last approach consist of considering simultaneously PR and Doppler measurements. The vector $\xi_{1}={\left(e_{r},{\dot{e}}_{r},n_{r},{\dot{n}}_{r},u_{r},{\dot{u}}_{r},\delta_{t},{\dot{\delta }}_{t}\right)}^{⊺}$ gathers the parameters involved in Equations (1) and (2). Having linearized Equations (1) and (2), the resolution of the derived system (having at least eight equations) can be achieved using the Gauss–Newton iterative algorithm. Specifically, if $X_{S}$ and ${\dot{X}}_{S}$ denote the matrices gathering the vectors $x_{S_{j}}$ and ${\dot{x}}_{S_{j}}$, respectively, and the increment $\delta {\hat{\xi_{1}}}^{\left(k\right)}$ to sum to previous estimate $\xi_{1}^{\left(k-1\right)}$ (*k* being the iteration number) is:

(4) $$\delta {\hat{\xi_{1}}}^{\left(k\right)}=\underset{\delta \xi_{1}}{\arg\min}∥\left(z-z^{\left(k-1\right)}\right)-H\left(X_{S},{\dot{X}}_{S},\xi_{1}^{\left(k-1\right)}\right)\delta \xi_{1}∥$$

where $z={\left(\rho_{1}\cdots \rho_{n},{\dot{\rho }}_{1}\cdots {\dot{\rho }}_{n}\right)}^{⊺}$ is the vector of observations, $z^{\left(k-1\right)}$ is the estimation of z computed from previous (iteration k-1) state vector $\xi_{1}^{\left(k-1\right)}$ and H is the Jacobian matrix of the Equations (1) and (2) system (see [29] for more details).

### 2.3. Dynamic Model

In order to increase the robustness of the estimation, this latter may be done considering not only one epoch, but several epochs. Then, the data acquired at the different epochs should be linked through a model. In [30], the authors propose a polynomial dynamic model fitted on a time interval, including multiple epochs. Limiting ourselves to the first order, PR measurements are related using a dynamic model involving GNSS, the receiver location and speed, so that the $\xi_{1}$ vector already defined is suitable. However, considering also Doppler measurements, the acceleration should be introduced in the dynamic model, and the considered state vector becomes $\xi_{2}={\left(e_{r},{\dot{e}}_{r},{\ddot{e}}_{r},n_{r},{\dot{n}}_{r},{\ddot{n}}_{r},u_{r},\dot{u_{r}},\delta_{t},{\dot{\delta }}_{t}\right)}^{⊺}$.

State vectors, either $\xi_{1}$ or $\xi_{2}$, at different instants are linearly linked through transition matrices $M_{i,\mathrm{dt}}$ of the considered dynamic models, defined as follows:

$$\begin{array}{ccc} C_{\mathrm{dt}} & = & \left(\begin{array}{cc} 1 & dt\\ 0 & 1 \end{array}\right),0_{2\times 2}=\left(\begin{array}{cc} 0 & 0\\ 0 & 0 \end{array}\right)\\ D_{\mathrm{dt}} & = & \left(\begin{array}{cc} \frac{dt^{2}}{2} & 0\\ dt & 0 \end{array}\right)I_{2\times 2}=\left(\begin{array}{cc} 1 & 0\\ 0 & 1 \end{array}\right) \end{array}$$

(5) $$\begin{array}{ccc} M_{1,\mathrm{dt}} & = & \left(\begin{array}{cccc} C_{\mathrm{dt}} & 0_{2\times 2} & 0_{2\times 2} & 0_{2\times 2}\\ 0_{2\times 2} & C_{\mathrm{dt}} & 0_{2\times 2} & 0_{2\times 2}\\ 0_{2\times 2} & 0_{2\times 2} & C_{\mathrm{dt}} & 0_{2\times 2}\\ 0_{2\times 2} & 0_{2\times 2} & 0_{2\times 2} & C_{\mathrm{dt}} \end{array}\right) \end{array}$$

(6) $$\begin{array}{ccc} M_{2,\mathrm{dt}} & = & \left(\begin{array}{cccccc} C_{\mathrm{dt}} & D_{\mathrm{dt}} & 0_{2\times 2} & 0_{2\times 2} & 0_{2\times 2}\\ 0_{2\times 2} & I_{2\times 2} & D_{\mathrm{dt}}^{\tau } & 0_{2\times 2} & 0_{2\times 2} & \\ 0_{2\times 2} & 0_{2\times 2} & C_{\mathrm{dt}} & 0_{2\times 2} & 0_{2\times 2}\\ 0_{2\times 2} & 0_{2\times 2} & 0_{2\times 2} & C_{\mathrm{dt}} & 0_{2\times 2}\\ 0_{2\times 2} & 0_{2\times 2} & 0_{2\times 2} & 0_{2\times 2} & C_{\mathrm{dt}} \end{array}\right) \end{array}$$

where the superscript ${}^{\tau }$ denotes the anti-diagonal transpose operator (the transpose of the matrix with respect to the anti-diagonal). Denoting $\xi_{i,t}$, $i\in \left\{1,2\right\}$, the state vector at *t*,

(7) $$\xi_{i,t+\mathrm{dt}}=M_{i,\mathrm{dt}}\xi_{i,t}+o\left(dt^{i+1}\right)$$

where $o\left(dt^{i+1}\right)$ is the error (approximation) of the considered dynamic model.

Using the dynamic Model Equation (7), we are now able to compute the expected measurements (PR or Doppler) at different instants. Specifically, denoting $T=\left\{t_{k}=t+kdt,k\in \left\{0,\cdots ,n_{ep}-1\right\}\right\}$ the set of epochs considered for the estimation of the solution, $X_{S_{i},t_{k}}={\left(e_{S_{i,t_{k}}},n_{S_{i,t_{k}}},u_{S_{i,t_{k}}}\right)}^{⊺}$ the satellite $S_{i}$ location at instant $t_{k}$ and the expected pseudo-range $\tilde{\rho_{i}}\left(t_{k}\right)$ from $S_{i}$ at $t_{k}$ may be derived from $\xi_{1}$:

(8) $$\begin{array}{ccc} \tilde{\rho_{i}}\left(t_{k}\left|\xi_{1,t}\right.\right) & = & ∥{\left[M_{1,\mathrm{kdt}}.\xi_{1,t}\right]}_{1,3,5}-X_{S_{i},t_{k}}∥+c\left(\delta_{t}-{\dot{\delta }}_{t}kdt\right) \end{array}$$

where the subscript in matrix notation ${\left[\;\right]}_{l_{1},l_{2},l_{3}}$ denotes the restriction of the matrix or vector to rows $l_{1}$, $l_{2}$ and $l_{3}$ and $\left|\left|v\right|\right|$ is the norm of vector v.

In a similar way, the Doppler measurement expected at $t_{k}$ from $S_{i}$ may be derived from $\xi_{2}$:

(9) $$\begin{array}{ccc} a_{i,t_{k}} & = & -\frac{{\left[M_{2,\mathrm{kdt}}.\xi_{2,t}\right]}_{1,4,7}-X_{S_{i},t_{k}}}{∥{\left[M_{2,\mathrm{kdt}}.\xi_{2,t}\right]}_{1,4,7}-X_{S_{i},t_{k}}∥}\\ {\tilde{\dot{\rho }}}_{i}\left(t_{k}\left|\xi_{2,t}\right.\right) & = & \left({\left[M_{2,\mathrm{kdt}}.\xi_{2,t}\right]}_{2,5,8}-{\dot{X}}_{S_{i},t_{k}}\right)\cdot a_{i,t_{k}}-c{\dot{\delta }}_{t} \end{array}$$

Then, using classical regression, the state vector optimal values ${\hat{\xi }}_{1,t}$ and ${\hat{\xi }}_{2,t}$ are those minimizing the quadratic errors:

(10) $${\hat{\xi }}_{1,t}=\underset{\xi_{1,t}}{\arg\min}\sum_{t_{k}\in T}\sum_{i\in I\left(t_{k}\right)}{\left[\tilde{\rho_{i}}\left(t_{k}\left|\xi_{1,t}\right.\right)-\rho_{i}\left(t_{k}\right)\right]}^{2}$$

(11) $$\begin{array}{cc} {\hat{\xi }}_{2,t} & =\underset{\xi_{2,t}}{\arg\min}\sum_{t_{k}\in T}\sum_{i\in I\left(t_{k}\right)}\left[{\left[\tilde{\rho_{i}}\left(t_{k}\left|\xi_{2,t}\right.\right)-\rho_{i}\left(t_{k}\right)\right]}^{2}\right.+\beta \left.{\left[{\tilde{\dot{\rho }}}_{i}\left(t_{k}\left|\xi_{2,t}\right.\right)-{\dot{\rho }}_{i}\left(t_{k}\right)\right]}^{2}\right] \end{array}$$

where $I\left(t_{k}\right)$ is the set of the indices of the satellites providing measurements at $t_{k}$ and *β* is a weighting factor between the residues associated with PR and Doppler data, respectively.

In previous equations, the minimization is performed considering all of the measurements (PR and/or Doppler ones) available for the considered set of epochs T. However, some of these measurements may correspond to outliers, and might then bias the estimation. In the following part the paper, in addition to the acronym PR, we use the abbreviations “Dp” for “Doppler measurement” and “(PR,Dp)” for “both PR and Doppler measurements”.

## 3. Proposed Approach

In the presence of outliers, several strategies have been proposed. Robust methods aim at automatically mitigating the weight of these outliers in the estimation. For instance, PF or its variants belonging to the class of robust estimators can theoretically cope with outliers simply by giving a very small weight to the generated particles. However, if this filter has proven its efficiency against noise, we will see that too many outliers jeopardize the filter stability. Then, in the case of GPS data processing, some statistical tests have been proposed to detect the outliers, e.g., [31]. The most simple to cope with these outliers is simply to discard them from the data measurements (just as if the corresponding satellites were blocked). This is the strategy of the standard Fault Detection and Exclusion (FDE) technique implemented in the GPS receivers (even if they can only cope with at most one erroneous measurement [32]). More sophisticated strategies have also been proposed, e.g., [15,33], that aim at correcting the outliers. However, in this study, we do not consider such strategies, because we focus on the following basic main questions:

- For the localization problem, are Doppler measurements less subject to outliers than PR measurements?
- Does the presence of outliers also impact robust localization algorithms, such as PF or the Rao–Blackwell Particle Filter?
- In the affirmative case, is it worth detecting and discarding these outliers?

Then, in the localization algorithm, we add an outlier detection step that will select the data (among those available) involved in the location estimation. Specifically, considering filtering algorithms with two steps, prediction and estimation, the outlier detection step is inserted before the estimation step.

Outliers are searched either only in the PR dataset or also in the Dp dataset, depending on the assumption on Dp robustness:

- If Doppler measurements are assumed reliable like in [1], they are directly used to derive ${\dot{x}}_{r}$, and the outlier detection is performed only within the PR set.
- Otherwise, we assume like [7] that, even if the Doppler measurements are less distorted by NLOS reception than PR measurements (and thus, more reliable), both Doppler and PR observations are contaminated by multipaths. Then, the outlier detection is applied for (PR,Dp), so that only Dp inliers are used to derive ${\dot{x}}_{r}$ (and only PR inliers are considered for the estimation step further).

### 3.1. Outlier Detection

The outlier detection is performed using the *a contrario* approach that we proposed in [23] extended to the case of (PR,Dp). The *a contrario* approach detects the inliers as observations that are “too regular to occur by chance”. “Chance” is measured through the Number of False Alarms (NFA), based on two items: a model, called the “naive” model, that represents the statistics of the outliers (the $H_{0}$ hypotheses in statistical decision theory) and a measurement that will allow the distinction of inlier and outlier sets under the “naive” model assumption. In [23], we have proposed and compared two “naive” models leading to two NFA criteria for partitioning the data between inliers and outliers. However, these models only deal with PR measurements. In this study, we extend to (PR,Dp) the first NFA criterion that experimentally leads to slightly better results than the second NFA criterion.

Before presenting the extended algorithm, let us specify the equations used and the notations. Assuming a value of $\xi_{i}$ denoted $\tilde{\xi_{i}}$ and the satellite features (location and velocity), we are able to compute using Equations (8) and (9) the expected value of PR or Doppler measurement. Then, we can compare these expected ones to the actually observed ones. By definition, the residues are the differences between computed measurements (under the $\tilde{\xi_{i}}$ hypothesis) and the observed ones: $r_{i}\left(t_{k}\right)$ associated with the PR observation at $t_{k}$, $\rho_{i}\left(t_{k}\right)$, is:

(12) $$r_{i}\left(t_{k}\right)=\left[{\tilde{\rho }}_{i}\left(t_{k}|\xi_{i}\right)-\rho_{i}\left(t_{k}\right)\right]$$

where ${\tilde{\rho }}_{i}\left(t_{k}|\xi_{i}\right)$ is computed using Equation (8), and ${\dot{r}}_{i}\left(t_{k}\right)$ associated with Doppler observation at $t_{k}$, ${\dot{\rho }}_{i}\left(t_{k}\right)$, is:

(13) $${\dot{r}}_{i}\left(t_{k}\right)=\left[{\tilde{\dot{\rho }}}_{i}\left(t_{k}|\xi_{i}\right)-{\dot{\rho }}_{i}\left(t_{k}\right)\right]$$

where ${\tilde{\dot{\rho }}}_{i}\left(t_{k}|\xi_{i}\right)$ is computed using Equation (9).

In order to give the same weight to both kinds of measurements, PR and Doppler ones, $r_{i}\left(t_{k}\right)$ and ${\dot{r}}_{i}\left(t_{k}\right)$ are normalized by their standard deviation, $\sigma_{PR}$ and $\sigma_{Dp}$, respectively, and gathered into vector R:

(14) $$R=(\frac{r_{1}\left(t_{n_{ep}-1}\right)}{\sigma_{PR}},\cdots ,\frac{r_{m}\left(t\right)}{\sigma_{PR}},\frac{{\dot{r}}_{m+1}\left(t_{n_{ep}-1}\right)}{\sigma_{Dp}},\cdots ,\frac{{\dot{r}}_{M}\left(t\right)}{\sigma_{Dp}})$$

with *M* the cardinality of (PR,Dp) set.

As for Equations (10) and (11), several epochs are considered. This allows us to increase the number of available data, as well as the quality of the estimation, provided that the dynamic model (Equations (8) and (9)) used to ‘align’ the data acquired at different epochs is sufficiently accurate. The number of considered epochs, $n_{ep}$, is then a compromise between data availability and dynamic model approximation. In the following, $S_{PR}$ and $S_{Dp}$ denote the sets of available observations (PR and Doppler measurements, respectively) over the considered interval of epochs T.

Let us now consider a subset of measurements noted D in the whole set of observations $\left\{S_{PR},S_{Dp}\right\}$. Given D and R (Equation (14)), $\delta_{D}^{2}$ is defined as the sum of the squares of R components for indices *j* belonging to D (indeed, $\left\{S_{PR},S_{Dp}\right\}$ measurements being indexed, D also corresponds to a set of indices). Then, according to [23], $\delta_{D}^{2}$ allows us to quantify the consistency of D through the NFA measure (associated with the Gaussian naive model $N\left(0,\sigma \right)$):

(15) $$NFA_{1}\left(D\right)=\eta_{1}\frac{1}{\Gamma \left(\frac{\left|D\right|}{2}\right)}\int_{0}^{\delta_{D}^{2}/2\sigma^{2}}e^{-t}t^{\frac{\left|D\right|}{2}-1}dt$$

where Γ is the Gamma function, $\left|.\right|$ is the cardinality set operator and $\eta_{1}$ is a normalization term that allows us to control the average number of false alarms [17].

The $\chi^{2}$ test using the SSE (Sum of Squared Error) is used in the classical RAIM (Receiver Autonomous Integrity Monitoring) method [10,34] to detect the presence of erroneous data. However, it requires an *a priori* parameter, namely the probability of false alarm $P_{FA}$, to threshold the values of SSE. Conversely, using the NFA criterion, we are free from the fitting of a threshold parameter, since the solution is derived by optimization of the NFA function: the subset of inliers is the subset of measurements that allows us to reach the minimal value of NFA. Let us underline the difference between the parameter *σ* involved in the naive model and a threshold parameter: whereas a set of inliers obtained by thresholding will be very sensitive to the used threshold value, we have shown [23] that the subset D that minimizes the NFA value is very robust to naive model parameter *σ*.

Algorithm 1 presents the extended version of Algorithm 1 of [23] that allows us to find the subset D minimizing the NFA criterion. Here, the input parameters are the observation sets $S_{PR}$ and $S_{Dp}$ (possibly empty if Doppler measurements are not considered), the number of iterations $N_{test}$, the parameter *σ* of naive model M, the standard deviations, $\sigma_{PR}$ and $\sigma_{Dp}$, for the residue normalization and the binary parameter $I_{Dp}$ that is equal to zero or one, depending on the kind of processed data: only PR data or (PR,Dp), respectively. The output parameters are the subset D of the inliers and the estimation of $\tilde{\xi_{i}}$.

Following the *a contrario* RANSAC principle (e.g., [35]), the algorithm performs different estimations or tests (loop until $N_{test}$) in order to select the best one according to the NFA criterion. Then, for each test, it performs the three following steps. First, the data selection step consists of randomly drawing d=8 elements in $S_{PR}$ (the set of PR observations) or, if $I_{Dp}$, d=10 elements in $S_{PR}$ and $S_{Dp}$ (the set of Doppler measurements). The numbers eight and 10 correspond to the minimum number of observations to estimate ${\tilde{\xi }}_{1}$ or ${\tilde{\xi }}_{2}$ further. $S_{PR}$ and $S_{Dp}$ include any available observations performed during the considered interval of $n_{ep}$ last epochs. According to [23], the random drawing of observations is biased in order to favor the drawing of favorable configurations of satellites. Since we use a sliding window over epochs, there is an overlapping between the sets of considered epochs for the estimation at two successive instants. Therefore, from the processing of the previous instant, we know the inliers corresponding to previous $n_{ep}-1$ epochs. Then, like in [23], random drawing is constrained, such that: (i) there is at least one measurement per epoch; (ii) for epochs before the last one, the PR Doppler measurements are chosen among the already detected inliers; (iii) the selection of different satellites is favored.

These *d* observations are used to derive a preliminary solution ${\tilde{\xi }}_{1}$ or ${\tilde{\xi }}_{2}$ (depending on the $I_{Dp}$ value). To derive this solution, a regularization term may be added to Equation (8) or Equation (9), allowing both better conditioning of the problem and the receiver trajectory being smoother. Considering the regularization term, instead of Equation (10), we have to solve Equation (16):

(16) $${\tilde{\xi }}_{1}=\underset{\xi_{1}}{\arg\min}\sum_{i\in \left\{1,\cdots ,d\right\}}{\left({\tilde{o}}_{i}\left(\xi_{1}\right)-o_{i}\right)}^{2}+{\lambda_{1}}^{⊺}\left[abs\left(\xi_{1}-\xi_{1,t\left|t-1\right.}\right)\right]$$

and instead of Equation (11), we have to solve Equation (17):

(17) $${\tilde{\xi }}_{2}=\underset{\xi_{2}}{\arg\min}\sum_{i\in \left\{1,\cdots ,\frac{d}{2}\right\}}\left[{\left(\tilde{o_{i}}\left(\xi_{2}\right)-o_{i}\right)}^{2}\right.+\beta \left.{\left({\tilde{\dot{o}}}_{i}\left(\xi_{2}\right)-{\dot{o}}_{i}\right)}^{2}\right]+{\lambda_{2}}^{⊺}\left[abs\left(\xi_{2}-\xi_{2,t\left|t-1\right.}\right)\right]$$

In Equation (16) and Equation (17), $\xi_{i,t\left|t-1\right.},i\in \left\{1,2\right\}$, is the predicted vector state according to dynamic Model (7); $abs\left(v\right)$ returns the vector of the absolute values of *v* components; and $\lambda_{i},i\in \left\{1,2\right\},$ is the vector of the regularization parameters (***λ*** weights the importance of the deviation between estimated ${\tilde{\xi }}_{i}$ and predicted state vector $\xi_{i,t\left|t-1\right.}$). The Appendix specifies the derivation of $\xi_{i,t\left|t-1\right.},i\in \left\{1,2\right\}$.


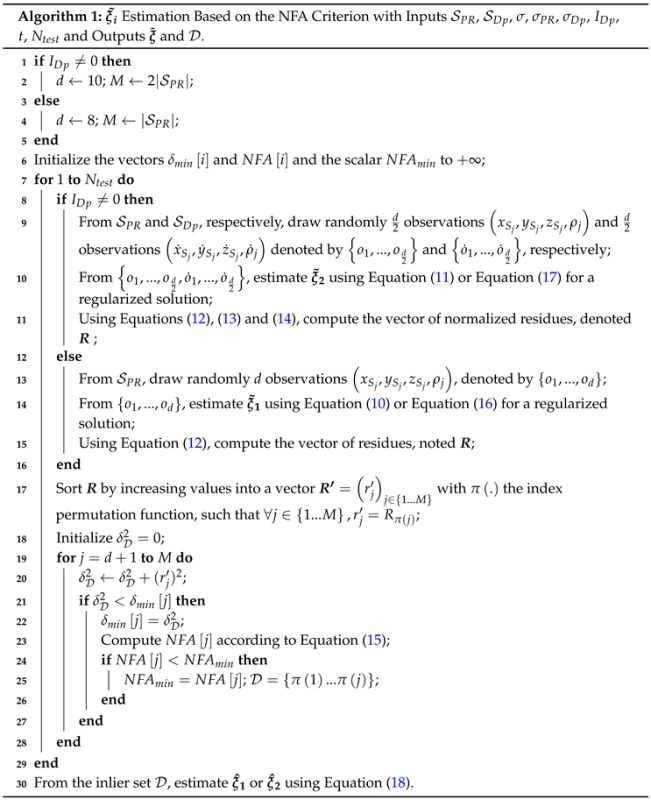


The second part of the algorithm computes the non-null residues for all of the other (not drawn) observations, either only PR or (PR,Dp). Having increasingly sorted the vector of residues, the last part of the algorithm computes the minimum NFA values by varying the cardinality of D (increasing from d+1 to *M*). $\delta_{min}\left[i\right]$ is a vector that stores the values of the minimal quadratic errors (sum of the squares of the residues) for every cardinality of subset D. Indeed, for a given cardinality of D, the NFAvalue is minimum for minimum value of quadratic error $d_{D}^{2}$ that is achieved considering the $\left|D\right|$ lowest values of residues (hence, the sorting of R). Then, $NFA_{min}\left[i\right]$ is a vector that stores the NFA values corresponding to $\delta_{min}\left[i\right]$; $NFA_{min}$ is the minimum among $NFA_{min}\left[i\right],\forall i\in \left\{d+1,\cdots ,M\right\}$. The inlier subset is the set D achieving the $NFA_{min}$ value. Finally, state vector ${\tilde{\xi }}_{1}$ or ${\tilde{\xi }}_{2}$ is estimated from D and Equation (18):

(18) $${\tilde{\xi }}_{i}=\underset{\xi_{i}}{\arg\min}\sum_{j\in D}R_{j}+{\lambda_{i}}^{⊺}\left[abs\left(\xi_{i}-\xi_{i,t\left|t-1\right.}\right)\right]$$

where $R_{j}$ is the residue provided by Equation (14).

Algorithm 1 has a linear complexity with $N_{test}$. For one iteration, the complexity mainly comes from state vector estimation (Algorithm A1, Appendix). The complexity of this latter depends on *d*: matrix inversion and matrix multiplication are in $O(d^{3})$. Then, the complexity of the sorting of R is in O(Mlog(M)). For NFA(PR,Dp), d=10, and *M* varies in $\left[12,33\right]$ considering a temporal window of three epochs. Therefore, to control the computation time, one should fit the parameter $N_{test}$.

Finally, note that, even if Algorithm 1 provides estimations of GNSS receiver localization parameters, the proposed coupling between Algorithm 1 and the robust localization algorithm (PF/RBPF presented in the next section) is only done in terms of data selection. Indeed, in Algorithm 1, the provided estimation only aims at evaluating the consistency of a subset of data, whereas PF/RBPF allows for non-linear/non-Gaussian data filtering that exploits some classic *a priori* parameteron the smoothness of the trajectories. Such an independence between the detection step (Algorithm 1) and the filtering step (PF/RBPF) increases the robustness of the global localization algorithm.

### 3.2. Localization Algorithm

The particle filter, also called the Sequential Monte Carlo (SMC) method, is a numerical method that consists of approximating the posterior probability $p(x_{t}|z_{t})$ (probability of the state $x_{t}$ given the set of observations $z_{t}$) using a sufficient number of particles $x_{t}^{i}$. A particle represents a state vector solution, and the associated weight $w_{t}^{i}$ represents its likelihood. Such a representation based on samples/particles allows us to approximate and deal with any statistical distribution of error, especially non-parametric ones and non-Gaussian ones.

#### 3.2.1. SIR-PF

The Sequential Importance Resampling (SIR) particle filter [13], also known as the “bootstrap filter”, is the most popular method to solve the non-linear filtering problem.

For SIR-PF, the number of the required particles is directly linked to the dimensionality of the state vector. In order to keep a reasonable number of particles (bounded to a few thousands), we assume that either the altitude is constant, as is often in urban environments, or it is known as in our case from the output $\tilde{\xi }$ of Algorithm 1, so that it has not been introduced in the state vector. For the same reasons, velocity is also excluded from the state vector (conversely to the RBPF state vector presented in the next section). Then, the SIR-PF particles are $x_{t}^{i}={\left(e_{t}^{i},n_{t}^{i},\delta_{t}^{i}\right)}^{⊺}$, where *i* denotes the particle index and *t* is the epoch.

At each epoch, the SIR-PF iterates the three steps “prediction”, “estimation” and “resampling”.

##### Prediction Step

This step, sometimes called PF time update, aims at providing an estimation of the state vector at the next time step. Note that if here, we place it at the beginning of iteration at time *t*, it can equivalently be placed at the end of iteration at t-1.

To predict the next position of the particle, we need an estimation of the velocity ${\dot{x}}_{r}$. Since, ${\dot{x}}_{r}$ is not part of the state vector, it should be provided by external data. Using GPS-only data, we consider Doppler measurements to derive ${\dot{x}}_{r}$: Doppler measurements at time t-1 provide PR rates from which we derive the receiver velocity ${\dot{x}}_{r}=\left({\dot{e}}_{r},{\dot{n}}_{r},{\dot{u}}_{r}\right)$ using Equation (3). In order to comply with common notations in the transportation and navigation community, ${\dot{x}}_{r}$ can be equivalently represented in terms of norm and orientation: $V_{t-1}^{i}=sqrt\left({\dot{e}}_{r}^{2}+{\dot{n}}_{r}^{2}\right)$ and $\theta_{t-1}^{i}=\arctan\left(\frac{{\dot{n}}_{r}}{{\dot{e}}_{r}}\right)$, respectively. Then, we predict the next state at *t* of the *i*-th particle according to:

(19) $$\left\{\begin{array}{ccc} e_{t}^{i} & = & e_{t-1}^{i}+V_{t-1}^{i}\cos\left(\theta_{t-1}^{i}\right)dt+\nu \left(\sigma_{e}\right)\\ n_{t}^{i} & = & n_{t-1}^{i}+V_{t-1}^{i}\sin\left(\theta_{t-1}^{i}\right)dt+\nu \left(\sigma_{n}\right)\\ \delta_{t}^{i} & = & \delta_{t-1}^{i}+{\dot{\delta }}_{t-1}dt+\nu \left(\sigma_{\delta_{t}}\right) \end{array}\right.$$

where the time step dt is equal to one and *ν* is the prediction noise associated with each component of the state vector. Indeed, as a stochastic approach, PF is based on stochastic simulations provided here by the addition (to the deterministic predictions state vectors) of a Gaussian noise with zero mean and standard deviation $\left(\sigma_{e},\sigma_{n},\sigma_{\delta_{t}}\right)$.

Note that in our case, the velocity used for prediction is estimated from $\left({\dot{e}}_{r},{\dot{n}}_{r}\right)$ at t-1. Instead of using Doppler measurements at t-1, we could have used those acquired at *t*. However, since the prediction is between t-1 and *t*, it will not provide necessarily a more accurate prediction. In comparison with the RBPF (presented in Section 3.2.2), let us underline that the velocity estimation is performed as an external process to the SIR-PF itself, since velocity is not a part of the state vector.

##### Estimation Step

This step, sometimes called PF measurement update, aims at correcting the prediction step estimate according to the observations. Since velocity is not represented in the state vector $x_{t}^{i}$, the posterior probability of our SIR-PF is only computed relatively to the PR measurements. It is denoted $p(\rho_{t}|x_{t}^{i})$ with $\rho_{t}$ the vector of $\rho_{i}$ observed at *t*.

The update process of weights $w_{t}^{i}$ is a weighting function of their previous values [36] by the observation likelihood function $p(\rho_{t}|x_{t}^{i})$: $w_{t}^{i}$∝$w_{t-1}^{i}p\left(\rho_{t}|x_{t}^{i}\right)$. In most cases, because of computational constraints, the likelihood function $p(\rho_{t}|x_{t}^{i})$ is approximated by a multivariate Gaussian density. Finally, normalization of the weights is performed so that $\sum_{i=1}^{k}w_{t}^{i}=1$.

Having updated the weights, the ‘optimal’ state vector ${\hat{x}}_{t}$ is derived as the weighted sum of all particles:

(20) $${\hat{x}}_{t}=\sum_{i=1}^{k}w_{t}^{i}x_{t}^{i}$$

##### Resampling

This step aims at preventing the degeneracy of the algorithm, in particular to avoid that computer resources are consumed by “unlikely” particles. During this step, a threshold is computed [36] to partition the set of the particles according to their weight [13]. Having removed the particles that present lower weight than the considered threshold, the remaining particles are duplicated in order to keep a constant number of particles, and all of the weights are reinitialized to a constant value (reciprocal of the total number of particles).

#### 3.2.2. Rao-Blackwellised PF

In previous PF, the velocity was estimated directly from Doppler measurements (being ‘outside’ of the PF estimation step, it does not take into account previous estimations of the PF prediction step). This boils down to assuming no noise on Doppler measurements. In order to avoid such an assumption and to be more realistic, we extend the state vector from ${\left(e,n,\delta_{t}\right)}^{⊺}$ to ${\left(e_{r},n_{r},\delta_{t},{\dot{e}}_{r},{\dot{n}}_{r},{\dot{\delta }}_{t},{\ddot{e}}_{r},{\ddot{n}}_{r},\right)}^{⊺}$, *i.e.*, its dimensionality increases from three to eight.

However, standard PF would require a very important number of particles to explore the whole space of solutions, and the PF would become intractable. On the other hand, the Rao-Blackwellization approach [37,38] was proposed both to reduce the complexity and to better approximate the solution in case of convex functions. It is based on the idea that splitting the state vector allows us to decrease the approximate error by exploiting linear substructures [25]. A classic case corresponds to the splitting of the initial state vector into two sub-vectors, one being estimated analytically and the other one by importance sampling (e.g., PF). Thus, the number of particles required for precise estimation remains tractable thanks to the lower dimensionality of the non-linear subsystem [25,38].

Considering our problem, we split the system of eight components describing the prediction step equations into two sub-systems, a linear and a non-linear one, as follows. The equations involving PR observations (Equation (1)) are non-linear leading to a non-linear system for deriving GPS position. On the other hand, the velocity estimation knowing the position of the receiver and the Doppler measurements is achieved solving a linear system (Equation (3)). Thus, we define the two state vectors $x_{\mathrm{pf}}={\left(e_{r},n_{r},\delta_{t}\right)}^{⊺}$ and $x_{\mathrm{kf}}={\left({\dot{e}}_{r},{\dot{n}}_{r},{\dot{\delta }}_{t},{\ddot{e}}_{r},{\ddot{n}}_{r}\right)}^{⊺}$.

The posterior probability of the RBPF is factorized:

(21) $$p\left(x_{\mathrm{kf},t},x_{\mathrm{pf},t}|z_{t}\right)=p\left(x_{\mathrm{kf},t}|x_{\mathrm{pf},t},z_{t}\right)p\left(x_{\mathrm{pf},t}|z_{t}\right)$$

where $z_{t}$ still denotes the set of observations. The first term is solved analytically using EKF, and the second term is estimated by Monte Carlo sampling using PF. Then, in RBPF, we can keep the same number of particles as in Section 3.2.1, while considering also the receiver velocity in the state vector and filtering it. The proposed model for RBPF is triangular:

(22) $$\begin{array}{c} \left(\begin{array}{c} x_{\mathrm{pf},t}\\ x_{\mathrm{kf},t} \end{array}\right)=\left(\begin{array}{cc} I_{3\times 3} & A_{\mathrm{pf},\mathrm{dt}}\\ 0_{5\times 3} & A_{\mathrm{kf},\mathrm{dt}} \end{array}\right)\left(\begin{array}{c} x_{\mathrm{pf},t-1}\\ x_{\mathrm{kf},t-1} \end{array}\right)+\left(\begin{array}{c} Q_{\mathrm{pf}}\\ Q_{\mathrm{kf}} \end{array}\right) \end{array}$$

where $I_{n\times n}$ is the square identity matrix of dimensionality *n*, $0_{m\times n}$ is the rectangular zero matrix of dimensionality m×n, $Q_{\mathrm{pf}}$ and $Q_{\mathrm{kf}}$ are the covariance matrices of the noise, which is assumed zero mean Gaussian (for notation shortness, we omitted the time dependency for covariance matrices) and $A_{\mathrm{pf},\mathrm{dt}}$ and $A_{\mathrm{kf},\mathrm{dt}}$ are the transition matrices defined as follows:

(23) $$\begin{array}{ccc} A_{\mathrm{pf},\mathrm{dt}} & = & \left(\begin{array}{ccccc} dt & 0 & 0 & \frac{dt^{2}}{2} & 0\\ 0 & dt & 0 & 0 & \frac{dt^{2}}{2}\\ 0 & 0 & dt & 0 & 0 \end{array}\right) \end{array}$$

(24) $$\begin{array}{ccc} A_{\mathrm{kf},\mathrm{dt}} & = & \left(\begin{array}{ccccc} 1 & 0 & 0 & dt & 0\\ 0 & 1 & 0 & 0 & dt\\ 0 & 0 & 1 & 0 & 0\\ 0 & 0 & 0 & 1 & 0\\ 0 & 0 & 0 & 0 & 1 \end{array}\right) \end{array}$$

The non-linear part is processed using the same PF presented in Section 3.2.1 to estimate the state vector of each particle $x_{\mathrm{pf}(i),t}$ and its associated weight $w_{t}^{i}$. The linear part is processed using an EKF applied to the state vector $x_{\mathrm{kf}(i),t}$ of each particle recursively. EKF involves two main steps:

##### Prediction Step

This step occurs between the prediction step and the estimation step of the SIR-PF. We define intermediate variables,

(25) $$\begin{array}{ccc} N_{t} & = & A_{\mathrm{pf},\mathrm{dt}}P_{t-1|t-1}A_{\mathrm{pf},\mathrm{dt}}^{⊺}+Q_{\mathrm{pf}} \end{array}$$

(26) $$\begin{array}{ccc} L_{t} & = & A_{\mathrm{kf},\mathrm{dt}}P_{t-1|t-1}A_{\mathrm{pf},\mathrm{dt}}^{⊺}N_{t}^{-1} \end{array}$$

(27) $$\begin{array}{ccc} y_{t} & = & x_{t}^{\mathrm{pf}}-x_{t-1}^{\mathrm{pf}} \end{array}$$

where $y_{t}$ is interpreted as an error measurement and $L_{t}$ and $N_{t}$ are intermediate matrices modeling the impact of the non-linear system on the linear estimation. Then,

(28) $$\begin{array}{ccc} {\hat{x}}_{\mathrm{kf},t|t-1} & = & A_{\mathrm{kf},\mathrm{dt}}{\hat{x}}_{\mathrm{kf},t-1|t-1}+L_{t}\left(y_{t}-\right.\left.A_{\mathrm{pf},\mathrm{dt}}{\hat{x}}_{\mathrm{kf},t-1|t-1}\right) \end{array}$$

(29) $$\begin{array}{ccc} P_{t|t-1} & = & A_{\mathrm{kf},\mathrm{dt}}P_{t-1|t-1}A_{\mathrm{kf},\mathrm{dt}}^{⊺}+Q_{\mathrm{kf}}-L_{t}N_{t}L_{t}^{⊺} \end{array}$$

where $P_{t-1|t-1}$ is the covariance matrix of $x_{t}^{\mathrm{kf}}$. Note that if the $A_{t}^{\mathrm{pf}}$ matrix is null, previous equations boil down to Kalman’s filter prediction step. Note that, since the prediction step presented in Section 3.2.1 is involved in Equation (27), the current prediction step occurs after the prediction of the non-linear part of RBPF.

##### Estimation Step

This step occurs between the estimation step and the resampling of the SIR-PF. It is the classical correction step of the extended Kalman filter.

(30) $$\begin{array}{ccc} {\hat{x}}_{\mathrm{kf},t|t} & = & {\hat{x}}_{\mathrm{kf},t|t-1}+K_{t}\left({\dot{\rho }}_{t}-C_{t}{\hat{x}}_{\mathrm{kf},t|t-1}\right) \end{array}$$

(31) $$\begin{array}{ccc} P_{t|t} & = & P_{t|t-1}-K_{t}C_{t}P_{t|t-1} \end{array}$$

(32) $$\begin{array}{ccc} K_{t} & = & P_{t|t-1}C_{t}^{⊺}{\left(C_{t}P_{t|t-1}C_{t}^{⊺}+R_{t}\right)}^{-1} \end{array}$$

where $C_{t}$ is the observation matrix of Doppler measurements derived from Equation (3).

This analytical correction of the ${\hat{x}}_{\mathrm{kf},t|t}$ subvector is independent from the estimation of ${\hat{x}}_{\mathrm{pf},t|t}$ that is performed according to the estimation step presented in Section 3.2.1 (Equation (20)).

One of the objectives of this study was to check the interest of removing outliers from the datasets, either PR or (PR,Dp). This can be achieved by comparing the localization results obtained using outlier detection coupled with PF or RBPF.

## 4. Experiment and Results

**Figure 1:**
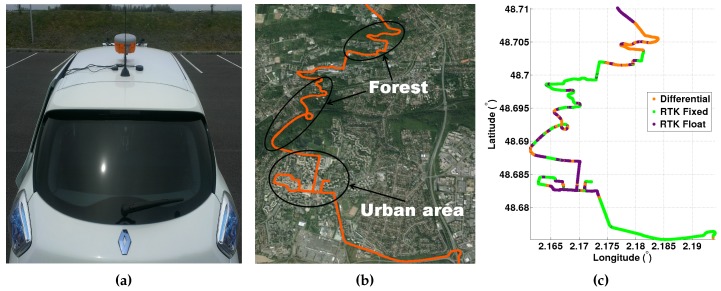
(**a**) Experimental platform with the three GPS visible on the roof of the car; (**b**,**c**) trajectory of the experiment, either (**b**) plotted on Google Earth${}^{©}$ or (**c**) labeled in terms of the quality of the Real-Time Kinematic (RTK) solution (“ground truth”).

In order to test our localization method, we have acquired data in constrained environments: an urban canyon and forest, characterized by NLOS reception. Figure 1b shows the receiver trajectory in the South of Paris (France). It is 5 km long for an experiment duration of 11 min.

### 4.1. Platform and Parameters

Figure 1a shows the used experimental vehicle ZOE that is equipped with two low cost GPS and one high cost GPS. The two low cost GPS are GARMIN 18x and UBLOX EVK-5T, which are single-frequency receivers delivering the positioning solution at 1 Hz. The high cost GPS is an APS-3 multi-frequency and multi-constellation receiver (L1/L2/L2C GPS, GLONASS and satellite-based augmentation system (SBAS)) that belongs to the GPS class RTK (Real-Time Kinematic). This latter has a sampling frequency equal to 1 Hz, and its location accuracy is equal to 1 cm, according to factory specifications in the case of the “fixed solution”. This solution was available during 41% of the experiment (*cf*. Figure 1c), whereas the two other solutions, ‘RTK float’ and ‘differential’, whose precision may drop until 40 cm, were available during 39% and 20%, respectively. APS-3 is used for two purposes: to establish the ground truth and to get the raw data used in the localization algorithms. However, the considered raw data are not post-processed by APS-3.

The GARMIN 18x has an accuracy (measured by the root mean square error) equal to 5 m in location and 0.05 m/s in velocity. Finally, the UBLOX EVK-5T acquires only PR measurements (no Doppler measurements) and is specified to have a location accuracy of 3 m in the static case and an open area.

**Figure 2:**
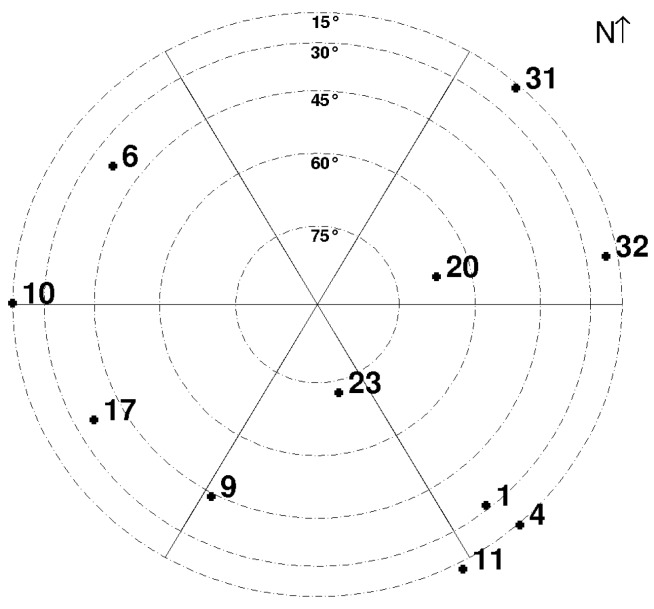
Skyplot configuration during the experimental data acquisition in the urban area.

The configuration of the satellites during the experiment is shown in Figure 2. The number of available satellites varies between four and 11 with an average equal to nine.

For the used algorithms, the parameters are:

- In Algorithm 1, $n_{ep}=3$, ${\lambda_{1}}^{⊺}=\left(\begin{array}{cccccccc} 200 & 20 & 200 & 20 & 10000 & 20 & 0 & 0 \end{array}\right)$ and ${\lambda_{2}}^{⊺}=\left(\begin{array}{cccccccccc} 20 & 2 & 20 & 20 & 2 & 20 & 10000 & 10000 & 0 & 0 \end{array}\right)$;
- In EKF, SIR-PF and RBPF, the PR precision is $\sigma_{PR}=5$ m, and the Dp precision is $\sigma_{Dp}=2$ Hz;
- In SIR-PF and RBPF, the number of particles is set to 3000.

### 4.2. Localization Results

The global performance of the localization is represented in terms of the cumulative distribution curve: the better the result, the greater the area below the cumulative distribution curve. In this study, we consider eleven localization processes. Two of them are GPS solutions themselves: either the UBLOX or the GARMIN GPS. The GARMIN and UBLOX EVK-5T solutions are plotted just as references, since it would be unfair to compare high cost and low cost GPS. However, we note that the GARMIN solution seems rather interesting, and even if the GARMIN algorithm is unknown, we may guess that it uses preprocessing of the measurements. For instance, if it uses the satellite elevation mask (discarding the satellites having an elevation lower than 15), according to Figure 2, the satellites $S_{4}$, $S_{10}$, $S_{11}$, $S_{31}$ and $S_{32}$ will not be used, which corresponds to frequent outliers, as we will see further.

The other processes correspond to different versions of the extended Kalman filter, the particle filter and the Rao-Blackwellised PF: without removing any outliers, by coupling it with the PR outlier detection or with the (PR,Dp) outlier detection. In the three filters (EKF, PF and RBPF), the initial solution is provided either by the least mean square solution or by the output of Algorithm 1 when there is an outlier detection step. For comparison, we also implement a recent robust outlier method called ORKF (Outlier Robust Kalman Filter) [39]. It is similar to the EKF, except that the covariance of the observation noise is estimated recursively inside the estimation step (releasing the assumption on the measurement precision).

**Figure 3:**
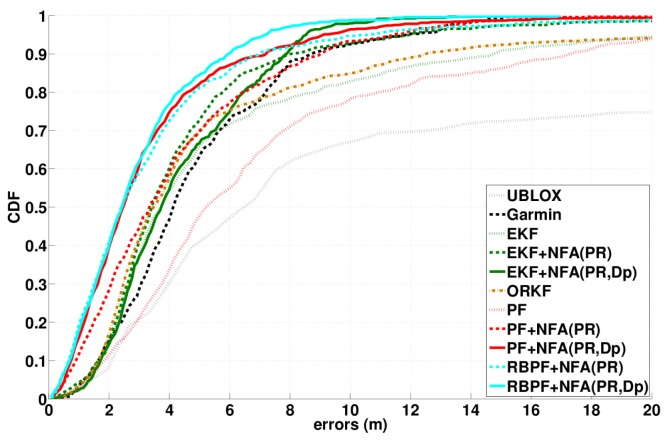
Cumulative distribution function of errors achieved by the four versions of KF, the five versions of particle filters and the two GPS solutions for our experiment of 11 min and 40 s.

Figure 3 and Table 1 allow us to draw the following conclusions:

- Among the implemented algorithms, the Particle Filter (PF) provides rather disappointing results with an error lower than 6 m in only 55% of cases. This relatively bad performance of PF, against EKF for instance, is probably due to the fact that the velocity is not part of the state vector; it is not at all filtered, conversely to the case of the EKF.
- The ORKF has better performance than the simplest version of PF and the classical EKF, and similar performance to EKF + NFA (PR) and EKF + NFA (PR + Dp) when the errors are less than 6 m.
- By removing the PR outliers at the entry of the filters, EKF + NFA (PR) and PF + NFA (PR) allow for much better localization than the ‘all-data’ EKF, PF or even ORKF for errors lower than 6 m. Besides, if EKF + NFA (PR) still performs better than PF + NFA (PR) for errors lower than 6 m, the gap has narrowed, and in terms of errors lower than 3 m, PF + NFA (PR) outperforms EKF + NFA (PR).
- By removing also the Dp outliers, PF + NFA (PR,Dp) provides better results than the previous methods. For instance, its 95th percentile corresponds to an error lower than 9 m, whereas PF + NFA (PR) percentile error is 11.5 m. This clearly illustrates the interest of removing also the Doppler outliers, especially as they are not filtered (by the estimation step of PF). Conversely, in the case of the EKF where velocities are filtered, the effect of removing Dp outliers is less clear: it appears just for errors lower than 9 m.
- By removing the PR outliers, RBPF + NFA (PR) has the same performance in localization as the PF + NFA (PR,Dp) version (see Table 1). This can be explained by the fact that, by filtering the velocity estimation, RBPF is rather robust to outliers in Doppler measurements. It also outperforms EKF + NFA (PR).
- Finally, removing also the Dp outliers, RBPF + NFA(PR,Dp) outperforms all of the other results. According to Table 1, if the performance for PR + NFA (PR,Dp) and the two RBPFs is close under 3 m, a higher level of confidence is achieved by RBPF + NFA (PR,Dp) for errors lower than 6 m and 9 m.

Table 2 shows the global precision of the localization. Precision was evaluated through three indicators: the $Norm_{1}$ norm, the $Norm_{2}$ and the mean and standard deviation of errors. $Norm_{1}$ and $Norm_{2}$ can be computed on east and north coordinates: precisely, denoting $\epsilon_{i}$ the error of the position at instant *i* along a given direction (east or north), $Norm_{1}=\sum_{i=1}^{n}\left|\epsilon_{i}\right|$ is the average of the absolute value of the errors, and $Norm_{2}=\sqrt{\sum_{i=1}^{n}\epsilon_{i}^{2}}$ is the root of average of the squared errors. Denoting $E_{i}$ the Euclidean distance between estimated and ‘ground truth’ positions at instant *i*, $\mu_{loc}$ and $\sigma_{loc}$ are the mean and the standard deviation of $E_{i}$ values. The results are consistent with Figure 3: Among the implemented algorithms, when using all-data, EKF and ORKF show good performance, and when removing the outliers (either PR or (PR,Dp)), RBPF outperforms the other approaches. The best results are obtained for NFA (PR,Dp) coupled with RBPF, even if the interest of removing outliers can also be noticed in the case of EKF or PF. Finally, to quantify the improvement due to the NFA outlier detection, we run RBPF with an elevation mask removing satellites below 15 (as is usually done on most GNSS receiver devices). The results are: $Norm_{1}=\left(2.82,3.10\right)$, $Norm_{2}=\left(4.68,4.38\right)$ and $\left(\mu_{loc},\sigma_{loc}\right)=\left(4.64,4.42\right)$. As expected, localization is less accurate than RBPF + NFA (PR,Dp) or even RBPF + NFA (PR), showing that the satellite elevation criterion does not exactly fit the outlier detection.

Table 3 shows the localization error computed on the three subparts of the trajectory corresponding to the three RTK solution qualities. The localization results are those obtained using RBPF with removal of outliers, either in the PR dataset or in the (PR,Dp) one (we focus on the best results), and the considered errors are computed as previously in terms of $Norm_{1}$, $Norm_{2}$ on east and north coordinates and the mean and standard deviation of the distance between estimation and ground truth. From Table 3, we observe a ‘correlation’ between the quality of the localization result and the RTK quality: localization is more precise on the RTK fixed part than on the RTK float part, and the differential part presents the worst localization results. There are two interpretations of such a fact: (i) the imprecision of the ground truth in the case of RTK float or the differential solutions introduces a supplementary error that slightly degrades the *estimated* precision of the localization; (ii) the RTK fixed solution occurs mainly in open areas (whereas the RTK float solution also occurs in an urban environment and the differential solution in the forest part; *cf*. Figure 1b and Figure 1c) where localization is generally good. Indeed, looking at the localization precision distribution *versus* the RTK solution for other methods , we also note that the results are more precise on the RTK fixed part of the trajectory.

### 4.3. Validation of the Outlier Estimation

In this section, we aim at checking the efficiency of Algorithm 1 in terms of outlier detection. The tricky part is the derivation of a ‘ground truth’ in terms of outliers. First of all, note that the definition of an outlier itself depends on the adopted point of view: from the statistical point of view, an outlier is a measurement *considerably dissimilar or inconsistent with the remainder of the data* [40], whereas from the physical point of view and according to the considered application, an outlier is then a measurement affected by multipath or NLOS reception. In this study, we adopt the statistical definition, and we derive an estimation of the biases, like in [7], as follows.

Among the (PR,Dp) set, we want to derive the subset of observations that behave as outliers from the statistical point of view. The only “ground truth” we have is the receiver position that is provided by the APS-3 GPS + GLONASS RTK. The construction of a “ground truth” about outliers from this ground truth about receiver localization proceeds in two steps: (i) firstly, estimation of the biases between observed measurements and expected ones; (ii) secondly, analysis of the biases to classify them as induced by outliers or by inliers.

#### 4.3.1. Bias Estimation for Qualitative Analysis

For the first step, we have to estimate the ‘expected’ measurements from the receiver localization ground truth. This latter allows us to derive the Euclidean distance between the satellite $S_{j}$ and the receiver position, $d_{j}$. However, we still need to estimate the clock bias ${\tilde{\delta }}_{t}$. In [7], the equation $c{\tilde{\delta }}_{t}=\frac{1}{N}\sum_{j=1}^{N}\left(\rho_{j}-d_{j}\right)$ was used. However, the mean estimator is not robust to outlier presence nor to the fact that the oscillator embedded on GPS receivers is not stable nor accurate. Then, we rather use the M estimator [41] as a simple solution among robust estimator class:

Assuming e the vector of residues of clock bias estimation ($e_{j}=\rho_{j}-d_{j}-c{\tilde{\delta }}_{t}$), $\alpha (e_{j})$ is the weight coefficient defined by $\alpha \left(e_{j}\right)=\frac{1}{\left|e_{j}\right|}$, and the optimal clock bias $c{\hat{\delta }}_{t}$ and the PR bias estimate $\tilde{\Delta }m_{j}$ are:

(33) $$\left\{\begin{array}{ccc} c{\hat{\delta }}_{t} & = & \frac{\sum_{j=1}^{N}\alpha \left(e_{j}\right)\left(\rho_{j}-d_{j}\right)}{\sum_{j=1}^{N}\alpha \left(e_{j}\right)}\\ \tilde{\Delta }m_{j} & = & \rho_{j}-d_{j}-c{\hat{\delta }}_{t} \end{array}\right.$$

**Figure 4:**
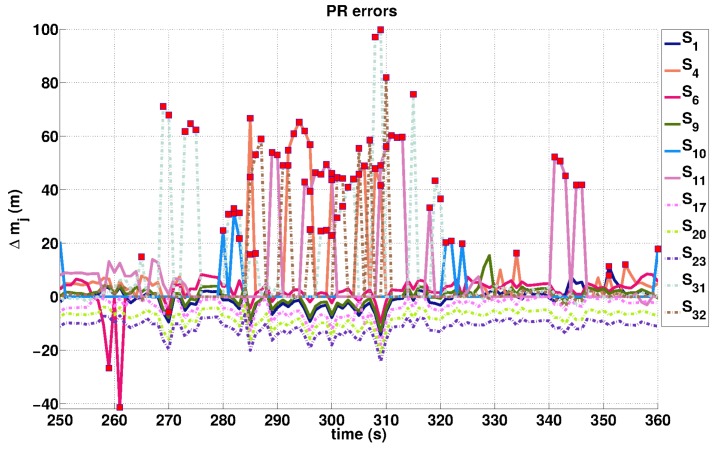
$\tilde{\Delta }m_{j}$ estimations on PR measurements acquired by the different satellites (numbered between 1 and 32). Red markers point out PR outliers detected by NFA.

**Figure 5:**
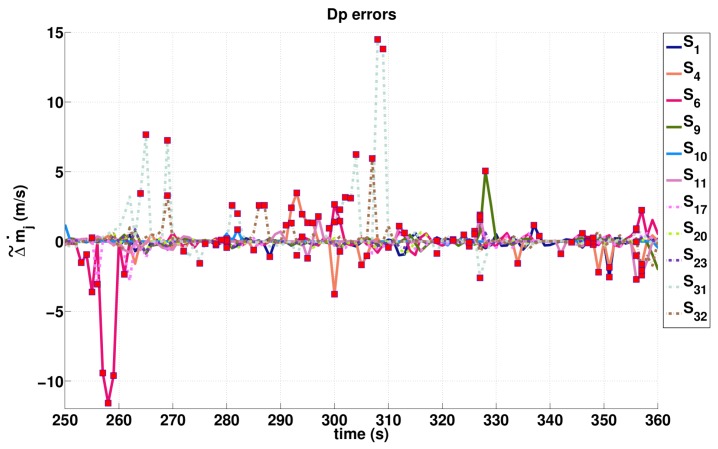
$\tilde{\Delta }{\dot{m}}_{j}$ estimations on Doppler measurements acquired by the different satellites (numbered between 1 and 32). Red markers point out Dp outliers detected by NFA.

In a similar way, we derive the biases $\tilde{\Delta }{\dot{m}}_{j}$ on Doppler measurements knowing both the velocity and location of the GPS receiver. Figure 4 and Figure 5 allow us to check qualitatively the consistency between the large biases (either in PR or Doppler measurements) and NFA detected outliers. Specifically, they show the temporal variation of the estimated biases for PR and Doppler observations of each satellite, and the values detected as outliers by the NFA algorithm are pointed out (by a red marker). We also observe that the estimated signal in Equation (33) is probably affected by atmospheric and electronic noises that differ from one satellite to another. This satellite specificity induces different biases even between consistent curves (e.g., see the $S_{17}$, $S_{20}$ and $S_{23}$ curves in Figure 4). In the Dp case, the estimation is less affected by atmospheric noise, so that the peaks in Figure 5 corresponding to potential NLOS reception or multipaths reception appear clearly.

#### 4.3.2. Bias Classification for Quantitative Analysis

To evaluate quantitatively the efficiency of outlier detection, we have to label the previously estimated biases either as (induced by) “outlier” or as “inlier”. Such a labeling was done only for the $\tilde{\Delta }m_{j}$ (due to the objective difficulty of labeling the $\tilde{\Delta }{\dot{m}}_{j}$) by a human operator as follows. For every epoch *t* of the experiment, a bias $\tilde{\Delta }m_{j}$ is labeled “outlier” if, at *t*, it appears neither consistent with the average bias of the considered satellite nor with the other satellite biases. Practically, a thresholding step relative to the median value of all $\tilde{\Delta }m_{j}$ at *t* is first applied (the test of consistency with other satellites), then followed by visual inspection of the selected biases. For instance, at time 351 s, even if $S_{23}$ presents a rather important $\tilde{\Delta }m_{j}$ value, only $S_{1}$ and $S_{4}$ are labeled as outliers. Even if previous labeling includes a subjective part, we assume that it is *statistically* unbiased, and we use it to analyze *statistically* outlier detection results.

From previous bias labeling, on the one hand, and inlier set D provided by Algorithm 1, we compute the numbers of True Positives (TP, PR ∈D with bias label “inlier”), false alarms called False Positives (FP, PR ∈D with bias label “outlier”), misdetections called False Negatives (FN, PR ∉D with bias label “inlier”) and True Negatives (TN, PR ∉D with bias label “outlier”). From these statistics, the accuracy $\frac{\left(TP+TN\right)}{\left(TP+TN+FP+FN\right)}$ and precision $\frac{\left(TP\right)}{\left(TP+FP\right)}$ are derived. The sample set includes 3498 PR samples corresponding to a sub-part of the experiment (of 7 mn ) where biases $\tilde{\Delta }m_{j}$ were labeled. Table 4 shows the obtained values. The two presented coupling ways between particle filter and outlier detection either restricted to the PR measurements or for (PR,Dp) are called “NFA (PR)” and “NFA (PR,Dp)”, respectively. By comparing these two approaches, we note that the performance of both of them is very high. Besides, they appear very close given the statistical imprecision and the labeling process.

#### 4.3.3. Correlation between PR and Doppler Outliers

Having, at least qualitatively, positively assessed the outlier detection, we can interpret its result also in terms of the occurrence of Doppler outliers.

In terms of global statistics and correlation between PR and Doppler outliers, during the experiment, NFA (PR) excludes 9.83% of available PR observations, whereas NFA (PR,Dp) discards less PR observations (8.37%), but discards 2.85% of available Doppler observations. Among the Doppler outliers, 54% are also PR outliers. Thus, one can deduce that, according to these statistics, in constrained environments, Doppler measurements present three-times less outliers than PR measurements, but they, nevertheless, suffer from NLOS or multipath phenomena.

## 5. Conclusions

In this paper, a new approach able to cope with a significant number of outliers was presented for GNSS positioning. Based on *a contrario* modeling, the Number of False Alarms (NFA) criterion allows us to partition the pseudo-range and Doppler measurements between inliers and outliers. Then, detected outliers are removed from the dataset to achieve robust estimation of receiver position and velocity. Two models based on particle filtering have been considered for the localization process. The first model (PF) only filters the receiver position, whereas the second model (RBPF) is a more complete filter that handles receiver position and velocity and using both PR and Doppler observations in its estimation step. Tests have been performed in the case of a receiver on board a vehicle traveling in urban canyons and forest areas. Results show that, by excluding erroneous measurements and filtering the noise of the observations, more accurate localization is achieved.

Future work will deal with the optimization of the time processing and memory. The *a contrario* approach may be parallelized, since the $N_{test}$ loop in Algorithm 1 may be run independently, and the comparison of the results to get the solution minimizing NFA may be only done at the end of the algorithm. Besides, the prediction part of the RBPF can also be processed simultaneously with the outlier detection. We will also investigate a stronger coupling between particle filtering and *a contrario* estimation. The proposed *a contrario* detection algorithm will not only provide the partition between inliers and outliers, but it could also provide an estimate of the state vector (used to interpret the measurements) that may be combined with the particle filter estimate in a fusion process. Finally, we aim at using a more sophisticated observation model instead of basic Equation (1), e.g., including the atmospheric effect, to analyze the detected outliers and, when possible, to correct them.

## Figures and Tables

**Table 1:** Percentiles of positioning errors. NFA, Number of False Alarms; PR, Pseudo-Range; Dp, Doppler measurement; RBPF, Rao–Blackwell Particle Filter; ORKF, Outlier Robust Kalman Filter.

Localization Method	% Error <3 m	% Error <6 m	% Error <9 m
UBLOX	20.9%	47.15%	64.92%
GARMIN	28.6%	72.97%	90.72%
EKF	37.26%	71.66%	80.75%
EKF + NFA (PR)	40.94%	81.82%	91.73%
EKF + NFA (PR,Dp)	37.13%	74.88%	96.49%
ORKF	40.83%	74.77%	83.22%
PF	21.1%	55.02%	75.12%
PF + NFA (PR)	44.6%	77.19%	89.85%
PF + NFA (PR,Dp)	59.95%	87.05%	94.61%
RBPF + NFA (PR)	61%	85.78%	93.38%
RBPF + NFA (PR,Dp)	61.96%	90.11%	98.28%

**Table 2:** Localization error (in m) on (east, north) coordinates, $Norm_{1}$ and $Norm_{2}$ of error, error mean and standard deviation: comparison of the four versions of KF, the five versions of particle filters and the two GPS solutions on our 11 min 40 s experiment.

Error Measure	Localization Algorithm	Data		
		All-Data	NFA (PR) Inliers	NFA (PR,Dp) Inliers
$L_{1}$	UBLOX	(11.92,10.20)	-	-
	GARMIN	(3.35,2.76)	-	-
	EKF	(3.76,4.50)	(2.63,3.18)	(3.31,2.24)
	ORKF	(3.55,4.31)	-	-
	PF	(6.68,6.72)	(2.61,2.83)	(1.82,2.41)
	RBPF	-	(1.84,2.69)	(1.62,2.17)
$L_{2}$	UBLOX	(20.44,18.60)	-	-
	GARMIN	(4.73,3.35)	-	-
	EKF	(5.77,7.47)	(3.43,5.00)	(3.92,3.09)
	ORKF	(5.55,7.79)	-	-
	PF	(9.09,9.49)	(3.48,3.86)	(2.95,3.51)
	RBPF	-	(3.37,3.53)	(2.51,3.20)
($\mu_{loc},\sigma_{loc}$)	UBLOX	(16.72,22.02)	-	-
	GARMIN	(4.91,3.08)	-	-
	EKF	(6.40,6.96)	(4.59,3.96)	(4.37,2.42)
	ORKF	(6.13,7.36)	-	-
	PF	(10.43,7.99)	(4.25,3.41)	(3.37,3.11)
	RBPF	-	(3.53,3.56)	(2.96,2.25)

**Table 3:** Proposed method localization error (in m) on (east, north) coordinates, $Norm_{1}$ and $Norm_{2}$ of error, error mean and standard deviation *versus* the quality of RTK solution used as the ground truth.

	Solution Quality	RBPF + NFA (PR)	RBPF + NFA (PR,Dp)
$L_{1}$	RTK fixed	(1.44,2.08)	(1.27,1.74)
	RTK float	(2.21,3.03)	(1.91,2.55)
	Differential	(2.16,4.16)	(2.03,2.62)
$L_{2}$	RTK fixed	(2.56,3.14)	(1.76,2.45)
	RTK float	(3.19,4.35)	(2.56,3.26)
	Differential	(3.70,6.02)	(2.75,3.85)
($\mu_{loc},\sigma_{loc}$)	RTK fixed	(2.74,2.99)	(2.38,1.85)
	RTK float	(4.05,3.57)	(3.44,2.31)
	Differential	(5.08,4.96)	(3.68,3.00)

**Table 4:** Performance of Algorithm 1 for outlier detection among PR measurements or (PR,Dp).

	*TP*	*FP*	*FN*	*TN*	Accuracy	Precision
NFA (PR)	3131	39	49	279	97.5	98.7
NFA (PR,Dp)	3112	91	45	250	96.1	97.2

## Appendix

Equation (18) is solved using Algorithm A1. It is presented in the case of $\xi_{2}$ estimation, but the case of $\xi_{1}$ may be derived as a specific case. The input data are the observations, for the previous solution $\xi_{\left(2,t|t-1\right)}$ and the regularization parameter vector ***λ***. Like in most practical applications, the regularization parameter is fitted (or learned) using a first set of data. In our case, we do not regularize clock bias and clock drift, $\delta_{t}$ and ${\dot{\delta }}_{t}$, so that the corresponding ***λ*** coefficients are set to zero. This algorithm involves the computation of the Jacobian that is as follows.

Let us define two non-linear functions:

(A1) $$f_{i}\left(X\right)=\sqrt{{\left(E_{r}-e_{S_{i}}\right)}^{2}+{\left(N_{r}-n_{S_{i}}\right)}^{2}+{\left(U_{r}-u_{S_{i}}\right)}^{2}}+c\Delta t$$

(A2) $$g_{i}\left(X\right)=\left({\dot{E}}_{r}-{\dot{e}}_{S_{i}}\right)a_{e_{i}}+\left({\dot{N}}_{r}-{\dot{n}}_{S_{i}}\right)a_{n_{i}}+\left({\dot{U}}_{r}-{\dot{u}}_{S_{i}}\right)a_{u_{i}}-c\dot{\delta }t$$

where $X^{⊺}=\left(e_{r},n_{r},u_{r},\delta t,{\dot{e}}_{r},{\dot{n}}_{r},{\dot{u}}_{r},\dot{\delta }t,{\ddot{e}}_{r},{\ddot{n}}_{r}\right)$ is the unknown state vector. Then:

$$\left\{\begin{array}{c} E_{r}=e_{r}+{\dot{e}}_{r}dt+{\ddot{e}}_{r}\frac{dt^{2}}{2},\\ N_{r}=n_{r}+{\dot{n}}_{r}dt+{\ddot{n}}_{r}\frac{dt^{2}}{2},\\ U_{r}=u_{r}+{\dot{u}}_{r}dt,\\ {\dot{E}}_{r}={\dot{e}}_{r}+{\ddot{e}}_{r}dt,\\ {\dot{N}}_{r}={\dot{n}}_{r}+{\ddot{n}}_{r}dt,\\ \Delta t=\delta t+\dot{\delta t}dt,\\ a_{e_{i}}=\frac{e_{S_{i}}-E_{r}}{R_{i}},\\ a_{n_{i}}=\frac{n_{S_{i}}-N_{r}}{R_{i}},\\ a_{u_{i}}=\frac{u_{S_{i}}-U_{r}}{R_{i}} \end{array}\right.$$

where dt is the time update and $R_{i}=\sqrt{{\left(E_{r}-e_{S_{i}}\right)}^{2}+{\left(N_{r}-n_{S_{i}}\right)}^{2}+{\left(U_{r}-u_{S_{i}}\right)}^{2}}$ is the distance receiver/satellite for all $S_{i},i\in \left\{1\cdots n\right\}$.

The Jacobian is:

(A3) $$J=\left(\begin{array}{cccc} \frac{\partial f_{1}\left(X\right)}{\partial e_{r}} & \frac{\partial f_{1}\left(X\right)}{\partial n_{r}} & \cdots & \frac{\partial f_{1}\left(X\right)}{\partial \;{\ddot{n}}_{r}}\\ \vdots & \vdots & \ddots & \vdots \\ \frac{\partial f_{n}\left(X\right)}{\partial e_{r}} & \frac{\partial f_{n}\left(X\right)}{\partial n_{r}} & \cdots & \frac{\partial f_{n}\left(X\right)}{\partial \;{\ddot{n}}_{r}}\\ \frac{\partial g_{1}\left(X\right)}{\partial e_{r}} & \frac{\partial g_{1}\left(X\right)}{\partial n_{r}} & \cdots & \frac{\partial g_{1}\left(X\right)}{\partial \;{\ddot{n}}_{r}}\\ \vdots & \vdots & \ddots & \vdots \\ \frac{\partial g_{n}\left(X\right)}{\partial e_{r}} & \frac{\partial g_{n}\left(X\right)}{\partial n_{r}} & \cdots & \frac{\partial g_{n}\left(X\right)}{\partial \;{\ddot{n}}_{r}} \end{array}\right)$$

where:

$$\left\{\begin{array}{c} \frac{\partial f_{i}\left(X\right)}{\partial e_{r}}=\frac{E_{r}-e_{S_{i}}}{R_{i}},\frac{\partial f_{i}\left(X\right)}{\partial n_{r}}=\frac{N_{r}-n_{S_{i}}}{R_{i}}\\ \frac{\partial f_{i}\left(X\right)}{\partial u_{r}}=\frac{U_{r}-u_{S_{i}}}{R_{i}},\frac{\partial f_{i}\left(X\right)}{\partial \delta t}=c\\ \frac{\partial f_{i}\left(X\right)}{\partial {\dot{e}}_{r}}=dt\frac{\partial f_{i}\left(X\right)}{\partial e_{r}},\frac{\partial f_{i}\left(X\right)}{\partial {\dot{n}}_{r}}=dt\frac{\partial f_{i}\left(X\right)}{\partial n_{r}}\\ \frac{\partial f_{i}\left(X\right)}{\partial {\dot{u}}_{r}}=dt\frac{\partial f_{i}\left(X\right)}{\partial u_{r}},\frac{\partial f_{i}\left(X\right)}{\partial \dot{\delta }t}=cdt,\\ \frac{\partial f_{i}\left(X\right)}{\partial {\ddot{e}}_{r}}=\frac{dt^{2}}{2}\frac{\partial f_{i}\left(X\right)}{\partial e_{r}},\frac{\partial f_{i}\left(X\right)}{\partial {\ddot{n}}_{r}}=\frac{dt^{2}}{2}\frac{\partial f_{i}\left(X\right)}{\partial n_{r}} \end{array}\right.$$

and:

$$\left\{\begin{array}{c} \frac{\partial g_{i}\left(X\right)}{\partial e_{r}}=\left({\dot{E}}_{r}-{\dot{e}}_{s}\right)\frac{\partial a_{e_{i}}}{\partial e_{r}}+\left({\dot{N}}_{r}-{\dot{n}}_{s}\right)\frac{\partial a_{n_{i}}}{\partial e_{r}}+\left({\dot{U}}_{r}-{\dot{u}}_{s}\right)\frac{\partial a_{u_{i}}}{\partial e_{r}}\\ \frac{\partial g_{i}\left(X\right)}{\partial n_{r}}=\left({\dot{E}}_{r}-{\dot{e}}_{s}\right)\frac{\partial a_{e_{i}}}{\partial n_{r}}+\left({\dot{N}}_{r}-{\dot{n}}_{s}\right)\frac{\partial a_{n_{i}}}{\partial n_{r}}+\left({\dot{U}}_{r}-{\dot{u}}_{s}\right)\frac{\partial a_{u_{i}}}{\partial n_{r}}\\ \frac{\partial g_{i}\left(X\right)}{\partial u_{r}}=\left({\dot{E}}_{r}-{\dot{e}}_{s}\right)\frac{\partial a_{e_{i}}}{\partial u_{r}}+\left({\dot{N}}_{r}-{\dot{n}}_{s}\right)\frac{\partial a_{n_{i}}}{\partial u_{r}}+\left({\dot{U}}_{r}-{\dot{u}}_{s}\right)\frac{\partial a_{u_{i}}}{\partial u_{r}}\\ \frac{\partial g_{i}\left(X\right)}{\partial \delta t}=0\\ \frac{\partial g_{i}\left(X\right)}{\partial {\dot{e}}_{r}}=a_{e_{i}},\frac{\partial g_{i}\left(X\right)}{\partial {\dot{n}}_{r}}=a_{n_{i}}\\ \frac{\partial g_{i}\left(X\right)}{\partial {\dot{u}}_{r}}=a_{u_{i}},\frac{\partial g_{i}\left(X\right)}{\partial \dot{\delta t}}=-cdt\\ \frac{\partial g_{i}\left(X\right)}{\partial {\ddot{e}}_{r}}=a_{e_{i}}dt,\frac{\partial g_{i}\left(X\right)}{\partial {\ddot{n}}_{r}}=a_{n_{i}}dt \end{array}\right.$$

The derivatives of the unit vector are given by:

$$\left\{\begin{array}{c} \frac{\partial a_{e_{i}}}{\partial e_{r}}=\frac{{\left(E_{r}-e_{s}\right)}^{2}-R_{i}^{2}}{R_{i}^{3}}\\ \frac{\partial a_{n_{i}}}{\partial n_{r}}=\frac{{\left(N_{r}-n_{s}\right)}^{2}-R_{i}^{2}}{R_{i}^{3}}\\ \frac{\partial a_{u_{i}}}{\partial u_{r}}=\frac{{\left(U_{r}-u_{s}\right)}^{2}-R_{i}^{2}}{R_{i}^{3}}\\ \frac{\partial a_{e_{i}}}{\partial n_{r}}=\frac{\partial a_{n_{i}}}{\partial e_{r}}=\frac{\left(E_{r}-e_{s}\right)\left(N_{r}-n_{s}\right)}{R_{i}^{3}}\\ \frac{\partial a_{e_{i}}}{\partial u_{r}}=\frac{\partial a_{u_{i}}}{\partial e_{r}}=\frac{\left(E_{r}-e_{s}\right)\left(U_{r}-u_{s}\right)}{R_{i}^{3}}\\ \frac{\partial a_{u_{i}}}{\partial n_{r}}=\frac{\partial a_{n_{i}}}{\partial u_{r}}=\frac{\left(U_{r}-u_{s}\right)\left(N_{r}-n_{s}\right)}{R_{i}^{3}} \end{array}\right.$$

The derivative of a relatively to the velocity and the acceleration are assumed null.

## References

[1] Kaplan E., Hegarty C. (2005). Understanding GPS: Principles and Applications.

[2] Skog I., Handel P. (2009). In-car positioning and navigation technologies: A survey. IEEE Trans. Intell. Transp. Syst..

[3] Chiang K.W., Duong T.T., Liao J.K. (2013). The Performance Analysis of a Real-Time Integrated INS/GPS Vehicle Navigation System with Abnormal GPS Measurement Elimination. Sensors.

[4] Peyraud S., Bataille D., Renault S., Ortiz M., Mougel F., Meizel D., Peyret F. (2013). About non-Line-of-Sight Satellite Detection and Exclusion in a 3D Map-Aided Localization Algorithm. Sensors.

[5] Lu W., Seignez E., Rodriguez F.A., Reynaud R. Lane marking based vehicle localization using particle filter and multi-kernel estimation. Proceedings of the 13th International Conference on Control Automation Robotics Vision (ICARCV).

[6] Mao X., Wada M., Hashimoto H. Nonlinear filtering algorithms for GPS using pseudorange and Doppler shift measurements. Proceedings of the IEEE 5th International Conference on Intelligent Transportation Systems.

[7] Le Marchand O., Bonnifait P., Ibanez-Guzmán J., Betaille D., Peyret F. (2009). Characterization of GPS multipath for passenger vehicles across urban environments. ATTI dell’Ist. Ital. Navig..

[8] Xie P., Petovello M. (2015). Measuring GNSS Multipath Distributions in Urban Canyon Environments. IEEE Trans. Instrum. Meas..

[9] Obst M., Wanielik G. Probabilistic non-line-of-sight detection in reliable urban GNSS vehicle localization based on an empirical sensor model. Proceedings of the IEEE Intelligent Vehicles Symposium (IV).

[10] Brown R.G. (1992). A baseline GPS RAIM scheme and a note on the equivalence of three RAIM methods. Navigation.

[11] Seignez E., Kieffer M., Lambert A., Walter E., Maurin T. (2009). Real-time bounded-error state estimation for vehicle tracking. Int. J. Robot. Res..

[12] Hewitson S., Wang J. (2007). GNSS Receiver Autonomous Integrity Monitoring with a Dynamic Model. J. Navig..

[13] Arulampalam M.S., Maskell S., Gordon N., Clapp T. (2002). A tutorial on particle filters for online nonlinear/non-Gaussian Bayesian tracking. IEEE Trans. Signal Process..

[14] Marais J., Nahimana D.F., Viandier N., Duflos E. (2013). GNSS accuracy enhancement based on pseudo range error estimation in an urban propagation environment. Expert Syst. Appl..

[15] Giremus A., Tourneret J.Y., Calmettes V. (2007). A particle filtering approach for joint detection/estimation of multipath effects on GPS measurements. IEEE Trans. Signal Process..

[16] Desolneux A., Moisan L., Morel J.M. (2000). Meaningful alignments. Int. J. Comput. Vis..

[17] Desolneux A., Moisan L., Morel J.M. (2007). From Gestalt Theory to Image Analysis: A Probabilistic Approach.

[18] Almansa A., Desolneux A., Vamech S. (2003). Vanishing point detection without any a priori information. IEEE Trans. Pattern Anal. Mach. Intell..

[19] Muse P., Sur F., Cao F., Gousseau Y., Morel J.M. (2006). An a-contrario Decision Method for Shape Element Recognition. Int. J. Comput. Vis..

[20] Burrus N., Bernard T.M., Jolion J.M. (2009). Image segmentation by a-contrario simulation. Pattern Recognit..

[21] Robin A., Moisan L., Le Hégarat-Mascle S. (2010). An a-contrario approach for subpixel change detection in satellite imagery. IEEE Trans. Pattern Anal. Mach. Intell..

[22] Ammar M., Le Hégarat-Mascle S., Vasiliu M., Reynaud R. (2013). An a-contrario approach for object detection in video sequence. Int. J. Pure Appl. Math..

[23] Zair S., Le Hégarat-Mascle S., Seignez E. (2016). A-contrario modeling for robust localization using raw GNSS data. IEEE Trans. Intell. Transp. Syst..

[24] Zair S., Le Hégarat-Mascle S., Seignez E. Coupling Outlier Detection with Particle Filter for GPS-Based Localization. Proceedings of the 2015 IEEE 18th International Conference on Intelligent Transportation Systems (ITSC).

[25] Schön T., Gustafsson F., Nordlund P.J. (2005). Marginalized particle filters for mixed linear/nonlinear state-space models. IEEE Trans. Signal Process..

[26] Rabaoui A., Viandier N., Duflos E., Marais J., Vanheeghe P. (2012). Dirichlet process mixtures for density estimation in dynamic nonlinear modeling: Application to GPS positioning in urban canyons. IEEE Trans. Signal Process..

[27] Gustafsson F., Gunnarsson F., Bergman N., Forssell U., Jansson J., Karlsson R., Nordlund P.J. (2002). Particle filters for positioning, navigation, and tracking. IEEE Trans. Signal Process..

[28] Zhang J., Zhang K., Grenfell R., Deakin R. (2006). GPS satellite velocity and acceleration determination using the broadcast ephemeris. J. Navig..

[29] Li L., Zhong J., Zhao M. (2011). Doppler-Aided GNSS Position Estimation with Weighted Least Squares. IEEE Trans. Veh. Technol..

[30] Zhou Z., Li B. (2015). GNSS windowing navigation with adaptively constructed dynamic model. GPS Solut..

[31] Knight N.L., Wang J. (2009). A comparison of outlier detection procedures and robust estimation methods in GPS positioning. J. Navig..

[32] Le Marchand O., Bonnifait P., Bañez-Guzmán J., Peyret F., Betaille D. Performance Evaluation of Fault Detection Algorithms as Applied to Automotive Localisation. Proceedings of the European Navigation Conference-GNSS 2008.

[33] Cheng C., Tourneret J.Y., Pan Q., Calmettes V. (2016). Detecting, estimating and correcting multipath biases affecting GNSS signals using a marginalized likelihood ratio-based method. Signal Process..

[34] Hewitson S., Wang J. (2006). GNSS receiver autonomous integrity monitoring (RAIM) performance analysis. GPS Solut..

[35] Rabin J., Delon J., Gousseau Y., Moisan L. MAC-RANSAC: A robust algorithm for the recognition of multiple objects. Proceedings of the Fifth International Symposium on 3D Data Processing, Visualization and Transmission (3DPTV 2010).

[36] Doucet A., de Freitas N., Gordon N. (2001). An Introduction to Sequential Monte Carlo Methods.

[37] Casella G., Robert C.P. (1996). Rao-Blackwellisation of sampling schemes. Biometrika.

[38] Doucet A., Godsill S., Andrieu C. (2000). On sequential Monte Carlo sampling methods for Bayesian filtering. Stat. Comput..

[39] Agamennoni G., Nieto J.I., Nebot E.M. An outlier-robust Kalman filter. Proceedings of the 2011 IEEE International Conference on Robotics and Automation (ICRA).

[40] Ramaswamy S., Rastogi R., Shim K. (2000). Efficient Algorithms for Mining Outliers from Large Data Sets.

[41] Huber P.J. (1964). Robust estimation of a location parameter. Ann. Math. Stat..

